# Investigating problematic uses of Facebook and other Internet activities among university students in Bangladesh during the COVID-19 pandemic

**DOI:** 10.1038/s41598-023-27394-w

**Published:** 2023-01-24

**Authors:** Abu Sayeed, Md. Saiful Islam, Enryka Christopher, Abdullah Al Zubayer, Satyajit Kundu, Mohammad Raihan Tariq, Mst. Sadia Sultana, Md. Hasan Al Banna, Md Hafizur Rahman, Md Shafiqul Islam Khan, M. Tasdik Hasan, Kamrun Nahar Koly

**Affiliations:** 1 Department of Post-Harvest Technology and Marketing, Patuakhali Science and Technology University, Patuakhali, Bangladesh; 2 Department of Public Health and Informatics, Jahangirnagar University, Savar, Dhaka Bangladesh; 3 Centre for Advanced Research Excellence in Public Health, Savar, Dhaka, Bangladesh; 4 Boston Children’s Hospital, Harvard Medical School, Boston, MA USA; 5 Department of Sociology, University of Barishal, Barishal, Bangladesh; 6 Department of Biochemistry and Food Analysis, Patuakhali Science and Technology University, Patuakhali, Bangladesh; 7 Department of Environmental Sanitation, Patuakhali Science and Technology University, Patuakhali, Bangladesh; 8 Department of Food Microbiology, Patuakhali Science and Technology University, Patuakhali, Bangladesh; 9 International Centre for Diarrhoeal Disease Research, Bangladesh (icddr,b), Mohakhali, Dhaka Bangladesh; 10 Action Lab, Department of Human Centred Computing, Faculty of Information Technology, Monash University, Melbourne, Australia; 11 Department of Public Health, State University of Bangladesh, Dhaka, Bangladesh; 12 Department of Primary Care & Mental Health, University of Liverpool, Liverpool, UK; 13 Global Health Institute, North South University, Dhaka, Bangladesh

**Keywords:** Human behaviour, Psychology, Risk factors

## Abstract

Problematic use of Internet (PUI) and problematic use of Facebook (PUF) has been linked to escalating behavioral health issues among university students and has increased during the COVID-19 pandemic. This study estimated the prevalence of and explored associated factors for PUI and PUF among Bangladeshi university students during the COVID-19 pandemic. A cross-sectional online survey was undertaken among 1101 Bangladeshi university students between November and December 2020. The Internet Addiction Test and Facebook Addiction Scale were used to assess PIU and PUF, respectively. A multiple linear regression analysis was performed to adjust for confounders. Among the participants, PUI and PUF were found in 39.3% and 37.1%, respectively. The multiple linear regression model indicated PUI was significantly associated with participants residing in a village, arts majors, those unsatisfied with their major, having mediocre parental relationships, failure in romantic relationships, physical comorbidities, longer use of the Internet, using the Internet for purposes other than education, using social media, and downloading movies/TV series. PUF was significantly associated with village residence, lower income, arts majors, failure in romantic relationships, longer use of the Internet, using the Internet for purposes other than education, and downloading movies/TV series. Both PUI and PUF have been prevalent among Bangladeshi university students during the COVID-19 pandemic. Longitudinal & exploratory studies are warranted in the future to identify causal factors for PUI and PUF and appropriate interventions should be designed quickly for this population.

## Introduction

The COVID-19 pandemic has led to increased usage of the Internet and social media^1^. Confinement and isolation due to social distancing measures have facilitated this increase in the usage of the Internet^2^. In addition, many individuals lost jobs or decided to make a career change due to the pandemic, and they are even more engaged with digital platforms to search for opportunities^2^. Facebook, one of the most accessed Internet applications, is useful for networking^3^. However, there is potential to misuse Facebook, leading to harms in psychological health, interpersonal connections, and academic or job performance^4^. Problematic use of Facebook (PUF), which is part of the more general problematic use of Internet (PUI), comprises an important area of research worldwide, as it explores negative social, financial, academic, work-related, physical, and psychological health impacts that are associated^5,6^.

Social media and Internet usage were relatively high even prior to the pandemic^7^. However, COVID-19 may have spurred on problematic or addictive usage for some people^8^. A meta-analysis conducted before the pandemic reported prevalence rates of PUI ranging from 0.8% to 26.7% globally^9^. Low and middle-income countries (LIMCs) had higher than average prevalence estimates, ranging from 14 to 55%^10^. Asian regions report the highest prevalence of PUI along with significant problems^10,11^. Previous studies have found smartphone addiction, Facebook addiction, depression, anxiety, making new friendships online, and getting into romantic relationships online to be factors associated with PUI ^12,13^. On the other hand, the prevalence of PUF specifically has been studied in the Turkey, Philippines, Malaysia, Peru, and Norway, and estimates vary from 4 to 43.2% ^14–16^. Previous studies have identified relationship issues, a history of domestic violence, stressful life events, sleep disturbances, and depression as potential risk factors for PUF^17^.

PUI and PUF are prevalent among young adults in Bangladesh. A recent study reported that about half of students surveyed had moderate to severe levels of PUI^12^. Another study suggested that 36.9% of students were at risk for PUF in Bangladesh^17^. As addictive behaviors formed during young adulthood are more likely to persist into adulthood, and appropriate interventions are needed now more than ever^18,19^.

The move to virtual education from in-person learning as a result of the pandemic has also increased other online activities among young adults, such as social media usage and online gaming^18,20^. Circumstances brought on by the COVID-19 pandemic has led to increases in stress, anxiety, depression, and post-traumatic stress^21,22^. Students, already using online spaces with more frequency, may turn to unhealthy virtual coping mechanisms to deal with their psychosocial health. Gambling, video gaming, binge-watching TV, utilizing social media, watching pornography, or surfing the Internet may temporarily ease stress, anxiety, or depression, yet can easily lead to more problems, including PUI and PUF^18^. In addition to that, PUI was predicted by gender, failure in love history, and stressful life event^23^, whereas PUF was predicted by having history of domestic violence, failure in love, and having stressful life events^17^.

Problematic behavior related to digital technology usage is exponentially increasing in developing countries, mirroring usage patterns of some developed countries^24^. Despite being a resource-poor setting, the government of Bangladesh is providing enormous support in the digitalization of the country^25–27^. As of July 2021, 46 million people in Bangladesh use Facebook, 46% of whom are aged between 18 and 24 years^28^. One study reported that one out of every two young Facebook users suffers from PUF^25^. Several studies investigated PUI and PUF in pre-COVID-19 Bangladesh^25–27^, but few studies on PUI have been conducted after the pandemic hit, and no study has yet focused on PUF in this new era. Many of these existing studies were limited by small sample sizes from non-representative populations, such as students of a specific university or medical college^25,29,30^. This study was conducted during a period which was entirely uncertain & a shock to everyone where the university students faced added challenges. The study aimed to find out the actual Internet and Facebook using behaviors of the university students to provide a context to the policy makers to design appropriate interventions to support the university students to address their problematic Internet and Facebook using behaviors during public health emergencies like this. Therefore, the gravity of this study is immense.

In Bangladesh, limited evidence are available on PUI and PUF, so contemporary findings are critically important to fill this research gap & informing the policy makers about the significance of the issue. Therefore, the current study aimed to investigate the prevalence of and factors associated with PUI and PUF among university students in Bangladesh.

## Materials and methods

### Study setting and participants

An online-based cross-sectional study was conducted among undergraduate and graduate university students from November 2020 to December 2020, around eight months after the onset of the COVID-19 pandemic in Bangladesh. Because a community-based, face-to-face survey was not feasible at that critical time, data were collected online via snowball sampling. A self-reported questionnaire was made accessible through an online survey link, and distributed it over several social media platforms (e.g., Facebook, WhatsApp, etc.). Inclusion criteria were Bangladeshi adults (≥ 18 years old), living in Bangladesh during the government-imposed lockdown, university students, and able to read and interpret Bengali.

### Sampling procedure

Sample size was estimated based on a single population proportion formula with the assumption of 95% confidence level (1.96) and 3% margin of error (0.03)^31^ to account for the two primary outcomes, PUI and PUF. Two different sample sizes for two outcomes were calculated for this study. For PUI, a 44% prevalence of PUI in a recent study among university students^23^ was used to calculate the needed sample size of 1,052. For PUF, a 36.9% prevalence of PUF in a recent study among university students^17^ was used to calculate the needed sample size of 994. A total of 1127 people took part in the survey. Twenty-six participants were excluded during data cleaning due to incomplete and inconsistent responses, leaving 1101 participants for the analysis.

### Survey procedure

Two bilingual researchers translated the questionnaire into Bengali from English. Another bilingual researcher back translated the questionnaire to ensure consistency and eliminate errors. The questionnaire was first piloted among 50 participants to check for clarity and consistency. Test participants used the comment feature to flag wordings that were not clear, which helped us to amend questions and answers clearer and easier to comprehend. Links to the electronic questionnaire were then distributed in Bengali among various social media platforms, aiming to reach all nine administrative divisions of Bangladesh. Upon clicking on the link, participants were led to the first page of the study questionnaire, which included a study summary, procedures, a confidentiality agreement, and an informed consent form. Participants answered anonymously, and electronic data were kept secure, using encrypted data collection platforms and password-protected computers. The survey was conducted following the human involving guidelines (i.e., Declaration of Helsinki). The research protocol was reviewed and approved by the Research Ethical Committee (REC) of the Department of Food Microbiology, Patuakhali Science and Technology University, Bangladesh (Approval No: FMB: October 29, 2020:16).

### Measures

A total of 51 items were included in the questionnaire, which was divided into four sections: (i) sociodemographic characteristics, (ii) health, behavioral, and Internet-use variables, (iii) Internet Addiction Test (IAT), and (iv) Bergen Facebook Addiction Scale.

#### Sociodemographic characteristics

This section included fourteen questions about age, sex, residence (town, city, or village area), family type (nuclear or joint), monthly household income, study year, major subject (Engineering, Business, Arts, Science), satisfaction with major subject (yes, no, moderate), relationship status with parents (poor, moderate, good, not applicable), experience of domestic violence (yes or no), relationship status (single, in a relationship, married), failure in a romantic relationship (yes or no), previous history of physical or sexual abuse (yes or no).

The term “failure in a romantic relationship” refers to a circumstance in which a person's relationship with his/her partner has failed or ended. This term is quite culturally distinctive, and it most closely refers to, in Bangladeshi culture, a failed attempt to get engaged in a meaningful romantic relationship that would eventually lead to marriage. If they respond “yes,” they had a breakup, it means they had previously been in a mutual romantic relationship that had ended. A “no” response suggests that the person had never been in a relationship that ended. Therefore, they are still in their first relationship. The N/A choice included people who were not interested in dating or being in a relationship because of their age or did not believe in the traditional relationship paradigm. Failure to maintain a relationship in a society with high importance on marriage can lead to stress and other mental health difficulties^17^.

#### Health, behavioral, and Internet-use variables

Health-related data was obtained from questions about physical comorbidity (yes or no). Questions about tobacco smoking (yes or no) and history of substance use (yes or no) yielded behavioral and lifestyle-related data. To avoid response bias, explicit questions about substance usage were not asked as it is a stigmatized issue. Engagement in Internet use for educational purposes, online gaming, browsing YouTube, utilizing a chatroom, using social media (such as Twitter, Instagram), movie/ TV series download, online shopping, and frequency of Internet use were among the Internet-use variables.

#### Internet Addiction Test (IAT)

Young (1998) developed the Internet Addiction Test (IAT) as a self-report tool to assess PUI^32^. There are 20 Likert-type questions on this scale, ranging from 0 (*‘never’*) to 5 (*‘always’*). Individual answer scores of questions were added together to create total scores, ranging from 0 to 100. Participants were divided into two “internet-usage” categories based on their IAT scores, with those scoring less than 50 being classified as “non-PUI” and those scoring 50 or more as “PUI.” Methodology from previous studies among Bangladeshi university students was used to determine this cutoff^23,27^. Cronbach's alpha coefficient for the entire IAT score was 0.83 in this study, indicating good reliability.

#### Bergen Facebook addiction scale (BFAS)

The Bergen Facebook Addiction Scale (BFAS) was used to measure PUF^4^. This instrument is comprised of six items (e.g., *“Tried to cut down on the use of Facebook without success?”*), each of which is assessed on a 5-point Likert scale ranging from 1 (*‘very rarely’*) to (*‘very often’*). The total score ranged from 6 to 30. Although cutoff values were not employed in the original study, the authors used a cutoff score of ≥ 18 in the current study, using a conservative monothetic scoring scheme of 3 or above for each question. This cutoff score is similar to a previously conducted study on this topic among Bangladeshi university students^17,33^. The current study's Cronbach's alpha for this scale was 0.79.

### Statistical analysis

Statistical analyses were performed using two statistical software packages: SPSS (version 25.0) and STATA (version 13.0). Descriptive statistics (e.g., frequencies, percentages, means, standard deviations) were computed using the SPSS. Finally, bivariate and multiple linear regressions were performed using the STATA. While constructing multiple linear regression models, confounders were selected from the bivariate regression analysis, applying a *p*-value of less than 0.25^34,35^. The conventional threshold (*p*-value less than 0.05) was considered statistically significant in the multiple regression models.

### Ethical approval

The research protocol was reviewed and approved by the Research Ethical Committee (REC) of the Department of Food Microbiology, Patuakhali Science and Technology University, Bangladesh (Approval No: FMB: October 29, 2020:16).

## Results

### General profile of participants

A total of 1101 responses from university students (58.4% male; mean age: 22.63 ± 2.03 years) comprised the study sample. Of them, the majority were single (76.9%), resided in cities (48.0%), belonged to nuclear families (77.6%), and reported their monthly family income between 25,000–50,000 BDT (43.6%) (Table 1). There was a fairly equal distribution of students from 1st years to master’s level (13.3%–29.8%), the majority studied science (39.6%), and a sizeable minority were not satisfied with their study major (8.1%).

**Table 1:** Distribution of variables and regression analysis for PUI.

Variables	Overall	PUI	Unadjusted estimates					Adjusted estimates				
	n (%)	Mean (SD)	B	SE	t	β	*p*-value	B	SE	t	β	*p*-value
Age	22.63 ± 2.03		0.28	0.28	0.75	0.02	0.456	–	–	–	–	–
**Sex**												
Male	643 (58.4)	44.13 (18.66)	1.15	1.15	1.09	0.03	0.275	–	–	–	–	–
Female	458 (41.6)	42.88 (18.99)				Ref						
**Relationship status**												
In a Relationship	214 (19.4)	45.81 (19.23)	1.44	1.44	1.98	0.06	0.048	1.35	1.29	1.05	0.03	0.296
Married	40 (3.6)	45.53 (16.8)	3.04	3.04	0.84	0.03	0.399	0.88	2.74	0.32	0.01	0.748
Single	847 (76.9)	42.96 (18.75)				Ref					Ref	
**Residence**												
Town	257 (23.3)	47.05 (19.06)	1.57	1.57	2.40	0.08	0.017	0.80	1.46	0.55	0.02	0.585
City	529 (48.0)	42.14 (18.95)	1.33	1.33	− 0.86	− 0.03	0.392	− 4.38	1.33	− 3.29	− 0.12	0.001
Village	315 (28.6)	43.28 (18.03)				Ref					Ref	
**Family type**												
Nuclear	854 (77.6)	43.99 (18.75)	1.36	1.36	1.25	0.04	0.210	− 0.32	1.24	− 0.26	− 0.01	0.797
Joint	247 (22.4)	42.29 (18.97)				Ref					Ref	
**Monthly household income**												
25,000–50,000 BDT	480 (43.6)	44.75 (18.76)	1.28	1.28	1.45	0.05	0.147	− 0.01	1.19	0.00	< 0.01	0.997
> 50,000 BDT	224 (20.3)	42.42 (18.73)	1.57	1.57	− 0.31	− 0.01	0.760	− 2.89	1.50	− 1.93	− 0.06	0.054
< 25,000 BDT	397 (36.1)	42.9 (18.86)				Ref					Ref	
**Study year**												
2nd year	177 (16.1)	45.19 (18.51)	2.10	2.10	1.83	0.08	0.068	− 0.39	1.88	− 0.21	− 0.01	0.837
3rd year	230 (20.9)	42.68 (18.65)	1.99	1.99	0.67	0.03	0.503	− 1.73	1.80	− 0.96	− 0.04	0.337
4th year	328 (29.8)	44.2 (18.96)	1.87	1.87	1.53	0.07	0.127	− 0.12	1.72	− 0.07	< − .01	0.947
Masters	220 (20)	43.91 (19.31)	2.01	2.01	1.28	0.05	0.202	1.10	1.82	0.61	0.02	0.544
1st year	146 (13.3)	41.35 (18.21)				Ref					Ref	
**Study major**												
Engineering	146 (13.3)	44.5 (18.84)	1.80	1.80	0.91	0.03	0.362	1.26	1.60	0.78	0.02	0.433
Business	235 (21.3)	42.85 (18.42)	1.52	1.52	− 0.01	< − 0.01	0.995	1.75	1.36	1.28	0.04	0.199
Arts	284 (25.8)	44.93 (19.23)	1.43	1.43	1.44	0.05	0.150	2.83	1.30	2.17	0.07	0.030
Science	436 (39.6)	42.86 (18.71)				Ref					Ref	
**Satisfaction with major**												
Moderate	330 (30)	46.58 (17.81)	1.25	1.25	3.85	0.12	< 0.001	3.14	1.16	2.71	0.08	0.007
Poor	89 (8.1)	46.78 (19.45)	2.10	2.10	2.39	0.07	0.017	1.08	1.90	0.57	0.02	0.569
Good	682 (61.9)	41.76 (18.97)				Ref					Ref	
**Relationship with parents**												
Mediocre	126 (11.4)	49.23 (19.53)	1.76	1.76	3.80	0.11	< 0.001	4.15	1.64	2.53	0.07	0.012
Poor	25 (2.3)	58 (21.58)	3.76	3.76	4.11	0.12	< 0.001	6.76	3.45	1.96	0.05	0.054
Not applicable	15 (1.4)	39.4 (15.98)	4.83	4.83	− 0.65	− 0.02	0.517	− 5.29	4.35	− 1.22	− 0.03	0.224
Good	935 (84.9)	42.53 (18.39)				Ref					Ref	
**Domestic violence**												
Yes	123 (11.2)	46.22 (19.03)	1.80	1.80	1.64	0.05	0.102	0.55	1.67	0.33	0.01	0.742
No	978 (88.8)	43.28 (18.76)				Ref					Ref	
**Failure in a romantic relationship**												
Yes	417 (37.9)	46.77 (18.22)	1.16	1.16	4.39	0.13	< 0.001	2.36	1.07	2.21	0.06	0.027
No	684 (62.1)	41.68 (18.90)				Ref					Ref	
**Sexually abused**												
Yes	116 (10.5)	49.39 (19.07)	1.84	1.84	3.52	0.11	< 0.001	2.95	1.83	1.61	0.05	0.108
No	985 (89.5)	42.93 (18.66)				Ref					Ref	
**Physically abuses**												
Yes	153 (13.9)	47.54 (19.33)	1.63	1.63	2.79	0.08	0.005	− 0.48	1.68	− 0.29	− 0.01	0.775
No	948 (86.1)	42.97 (18.65)				Ref					Ref	
**Tobacco smoking**												
Yes	265 (24.1)	43.71 (18.48)	1.33	1.33	0.10	< 0.01	0.923	–	–	–	–	–
No	836 (75.9)	43.58 (18.91)				Ref						
**Drug use**												
Yes	49 (4.5)	48.96 (19.78)	2.74	2.74	2.04	0.06	0.041	2.51	2.48	1.01	0.03	0.312
No	1052 (95.5)	43.36 (18.73)				Ref					Ref	
**Physical comorbidity**												
Yes	191 (17.3)	49.03 (17.44)	1.48	1.48	4.42	0.13	< 0.001	4.54	1.37	3.32	0.09	0.001
No	910 (82.7)	42.47 (18.89)				Ref					Ref	
**Time spent per day using Internet**												
2–3 h	253 (23)	36.06 (14.89)	1.90	1.90	3.28	0.13	0.001	5.29	1.83	2.89	0.11	0.004
4–5 h	293 (26.6)	44.76 (17.1)	1.85	1.85	8.05	0.35	< 0.001	13.05	1.81	7.21	0.31	< 0.001
> 5 h	432 (39.2)	51.16 (18.66)	1.76	1.76	12.09	0.55	< 0.001	19.07	1.76	10.83	0.50	< 0.001
< 2 h	123 (11.2)	29.85 (16.86)				Ref					Ref	Ref
**Educational or information purpose**												
No	199 (18.1)	51.26 (20.49)	1.45	1.45	6.46	0.19	< 0.001	7.19	1.34	5.37	0.15	< 0.001
Yes	902 (81.9)	41.92 (17.99)				Ref					Ref	
**Use chatting site like Messenger, WeChat, WhatsApp etc**												
Yes	1025 (93.1)	44.09 (18.49)	2.23	2.23	3.14	0.09	0.002	3.21	2.17	1.48	0.04	0.140
No	76 (6.9)	37.09 (21.72)				Ref					Ref	
**Online gaming**												
Yes	347 (31.5)	44.53 (19)	1.22	1.22	1.10	0.03	0.270	–	–	–	–	–
No	754 (68.5)	43.18 (18.71)				Ref						
**YouTubing or watching videos**												
Yes	1039 (94.4)	43.88 (18.62)	2.46	2.46	1.98	0.06	0.048	1.53	2.32	0.66	0.02	0.509
No	62 (5.6)	39.03 (21.24)				Ref					Ref	
**Using social media like FB, Twitter, Instagram, etc**												
Yes	1037 (94.2)	44.16 (18.54)	2.41	2.41	3.97	0.12	< 0.001	4.80	2.35	2.05	0.06	0.041
No	64 (5.8)	34.61 (20.74)				Ref					Ref	
**Movie/ TV series downloading**												
Yes	708 (64.3)	45.59 (18.31)	1.17	1.17	4.74	0.14	< 0.001	3.13	1.08	2.90	0.08	0.004
No	393 (35.7)	40.04 (19.16)				Ref					Ref	
**Online shopping**												
Yes	632 (57.4)	43.74 (18.65)	1.15	1.15	0.27	0.01	0.787	–	–	–	–	–
No	469 (42.6)	43.43 (19.02)				Ref						

*SD* Standard deviations; *B* unstandardized regression coefficient; *SE* Standard error; *β* standardized regression coefficient.

Most reported a good relationship with their parents (84.9%), and 11.2% reported experiencing domestic violence. A sizable number of participants reported failure in a romantic relationship (37.9%), sexual abuse (10.5%), and physical abuse (13.9%). One-fourth of participants smoked cigarettes (24.1%), and 4.5% used illicit drugs. Additionally, 17.3% of participants reported that they had comorbidities.

Many used the Internet for more than five hours daily (39.2%), and it was mostly used for educational purposes (81.9%), chatting (Messenger, WeChat, WhatsApp, etc.; 93.1%), YouTubing or watching videos (94.4%), social media (FB, Twitter, Instagram; 94.2%), movie/ TV series downloading (64.3%), and online shopping (57.4%).

### Problematic use of Internet (PUI)

**Figure 1:**
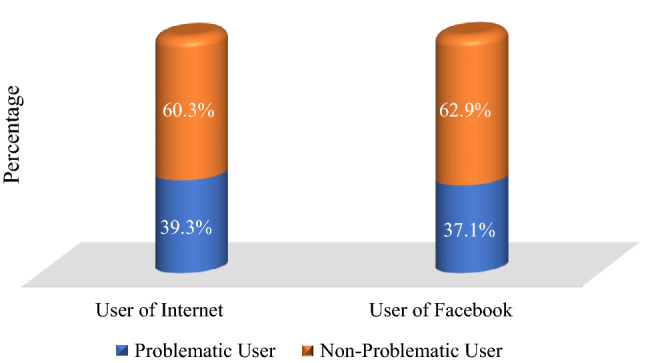
Prevalence of Problematic Use of the Internet (PUI) and Problematic Use of Facebook (PUF).

The prevalence of PUI was 39.3% among university students during the COVID-19 pandemic (Fig. 1). In a bivariate regression analysis the following were identified as candidate variables (*p* < 0.25) of the multivariable regression: relationship status, residence, family type, household income, study year, study major, satisfaction with major subject, relationship with parents, domestic violence, failure in romantic relationship, sexual abuse, physical abuse, drug addiction, physical comorbidities, frequency of Internet use, education or information Internet use, online chatting, YouTubing, social media, and video downloading. For the multiple linear regression model, the positively predicting factors of PUI included: (i) having arts major (β = 0.07, *p* = 0.030) in reference to ‘science’, (ii) having moderate satisfaction with their major (β = 0.08, *p* = 0.007) in reference to ‘good’, (iii) having mediocre relationship with parents (β = 0.07, *p* = 0.012) in reference to ‘good’, (iv) being failure in romantic relationship (β = 0.06, *p* = 0.027) in reference to ‘none’, (v) having physical comorbidities (β = 0.09, *p* = 0.001), (vi) having excessive Internet use (for 2–3 h [β = 0.11, *p* = 0.004], for 4–5 h [β = 0.31, *p* < 0.001], and for > 5 h [β = 0.50, *p* < 0.001]) in reference to ‘ < 2 h’, (vii) using Internet for purposes other than education (β = 0.15, *p* < 0.001), (viii) using social media (β = 0.06, *p* = 0.041), and (ix) downloading movies/TV series (β = 0.08, *p* = 0.004) (Table 1). The negatively predicting factors of PUI included: residing in city areas (β =  − 0.12, *p* = 0.001) in reference to ‘village’.

### Problematic use of Facebook (PUF)

The prevalence of PUF was 37.1% among university students during the COVID-19 pandemic (Fig. 1). Relationship status, residence, family type, household income, study year, study major, satisfaction with major subject, relationship with parents, domestic violence, failure in love, sexual abuse, physical abuse, physical comorbidities, frequency of Internet use, Internet use for education or information purposes, online chatting, and downloading movies/TV series were identified as candidate variables (*p* < 0.25) for the multivariable regression. For the multiple linear regression model, the positively predicting factors of PUF included: (i) having arts major (β = 0.07, *p* = 0.021) in reference to ‘science’, (ii) being failure in romantic relationship (0.06 = 0.027) in reference to ‘none’, (iii) having excessive Internet use (for 4–5 h [β = 0.27, *p* < 0.001], and for > 5 h/day [β = 0.50, *p* < 0.001]), (iv) using Internet for purposes other than education (β = 0.15, *p* < 0.001), and (v) downloading movies/TV series (β = 0.06, *p* = 0.031) (Table 2). The negatively predicting factors of PUF included: (i) residing in city areas (β =  − 0.10, *p* = 0.007) in reference to ‘village’, and (ii) having household income of more than 50,000 BDT (β =  − 0.09, *p* = 0.006) in reference to ‘ < 25,000 BDT’.

**Table 2:** Distribution of variables and regression analysis for PUF.

Variables	PUF	Unadjusted estimates					Adjusted estimates				
	Mean (SD)	B	SE	t	β	*p* − value	B	SE	t	β	*p* − value
**Age**		0.09	0.08	1.10	0.03	0.272	–	–	–	–	–
**Sex**											
Female	15.18 (5.69)	0.15	0.34	0.44	0.01	0.660	–	–	–	–	–
Male	15.33 (5.36)				Ref						
**Relationship status**											
In a Relationship	15.73 (4.98)	0.61	0.42	1.46	0.04	0.144	0.37	0.40	0.93	0.03	0.355
Married	15.92 (5.21)	0.81	0.89	0.91	0.03	0.363	0.58	0.84	0.69	0.02	0.491
Single	15.11 (5.63)				Ref					Ref	
**Residence**											
Town	15.93 (5.56)	0.49	0.46	1.07	0.04	0.286	− 0.11	0.45	− 0.25	− 0.01	0.801
City	14.83 (5.76)	− 0.61	0.39	− 1.56	− 0.06	0.119	− 1.10	0.41	− 2.69	− 0.10	0.007
Village	15.44 (4.94)				Ref					Ref	
**Family type**											
Nuclear	15.39 (5.53)	0.56	0.40	1.42	0.04	0.156	0.19	0.38	0.50	0.01	0.618
Joint	14.83 (5.4)				Ref					Ref	
**Monthly household income**											
25,000 − 50,000 BDT	15.5 (5.4)	0.11	0.37	0.28	0.01	0.777	− 0.25	0.36	− 0.68	− 0.02	0.493
> 50,000 BDT	14.54 (5.53)	− 0.85	0.46	− 1.85	− 0.06	0.064	− 1.26	0.46	− 2.75	− 0.09	0.006
< 25,000 BDT	15.39 (5.58)				Ref					Ref	
**Study year**											
2nd year	15.81 (5.58)	1.16	0.61	1.88	0.08	0.060	0.34	0.58	0.59	0.02	0.557
3rd year	14.93 (5.23)	0.28	0.58	0.49	0.02	0.625	− 0.22	0.55	− 0.39	− 0.02	0.695
4th year	15.59 (5.93)	0.94	0.55	1.73	0.08	0.085	0.40	0.53	0.76	0.03	0.447
Masters	15.08 (5.15)	0.43	0.59	0.73	0.03	0.463	0.19	0.56	0.34	0.01	0.730
1st year	14.65 (5.27)				Ref					Ref	
**Study major**											
Engineering	15.14 (5.43)	0.05	0.53	0.09	< .01	0.931	− 0.09	0.49	− 0.19	− 0.01	0.852
Business	15.02 (5.33)	− 0.08	0.45	− 0.18	− 0.01	0.855	0.37	0.42	0.88	0.03	0.381
Arts	15.78 (5.53)	0.68	0.42	1.63	0.05	0.104	0.93	0.40	2.32	0.07	0.021
Science	15.1 (5.59)				Ref					Ref	
**Satisfaction with major**											
Moderate	15.81 (5.4)	0.92	0.37	2.51	0.08	0.012	0.61	0.36	1.72	0.05	0.085
No	16.16 (5.58)	1.27	0.62	2.06	0.06	0.040	0.23	0.58	0.39	0.01	0.699
Yes	14.88 (5.51)				Ref					Ref	
**Relationship with parents**											
Mediocre	16.36 (5.42)	1.33	0.52	2.57	0.08	0.010	0.63	0.50	1.25	0.04	0.212
Poor	18.84 (5.83)	3.81	1.11	3.44	0.10	0.001	1.62	1.06	1.53	0.04	0.126
Not applicable	14.93 (7.16)	− 0.09	1.42	− 0.06	< − 0.01	0.948	− 0.43	1.34	− 0.32	− 0.01	0.747
Good	15.03 (5.43)				Ref					Ref	
**Domestic violence**											
Yes	16.15 (5.52)	0.99	0.53	1.89	0.06	0.059	0.63	0.51	1.24	0.04	0.216
No	15.15 (5.49)				Ref					Ref	
**Failure in romantic relationship**											
Yes	16.01 (5.6)	1.20	0.34	3.53	0.11	< 0.001	0.72	0.33	2.22	0.06	0.027
No	14.81 (5.39)				Ref					Ref	
**Sexually abuse**											
Yes	16.16 (5.76)	1.00	0.54	1.85	0.06	0.065	0.18	0.56	0.32	0.01	0.747
No	15.16 (5.46)				Ref					Ref	
**Physically abuse**											
Yes	15.8 (5.91)	0.63	0.48	1.31	0.04	0.190	− 0.33	0.51	− 0.64	− 0.02	0.523
No	15.18 (5.43)				Ref					Ref	
**Tobacco smoking**											
Yes	15.07 (5.15)	− 0.26	0.39	− 0.66	− 0.02	0.507	–	–	–	–	–
No	15.33 (5.61)				Ref						
**Drug use**											
Yes	16.02 (5.64)	0.79	0.80	0.99	0.03	0.325	–	–	–	–	–
No	15.23 (5.49)				Ref						
**Physical comorbidity**											
Yes	16.34 (5.63)	1.30	0.44	2.99	0.09	0.003	0.80	0.42	1.90	0.06	0.055
No	15.04 (5.45)				Ref					Ref	
**Time spent per day using Internet**											
2 − 3 h	13.25 (4.75)	1.07	0.57	1.87	0.08	0.062	0.89	0.56	1.59	0.07	0.112
4 − 5 h	15.69 (5.22)	3.51	0.56	6.28	0.28	< 0.001	3.31	0.55	5.97	0.27	< 0.001
> 5 h	17.04 (5.57)	4.86	0.53	9.14	0.48	< 0.001	4.53	0.54	8.41	0.40	< 0.001
< 2 h	12.18 (4.67)				Ref					Ref	
**Educational or information purpose**											
No	17.5 (5.66)	2.73	0.42	6.45	0.19	< 0.001	2.21	0.41	5.43	0.15	< 0.001
Yes	14.77 (5.34)				Ref					Ref	
**Use chatting site like Messenger, WeChat, WhatsApp etc**											
Yes	15.29 (5.47)	0.40	0.65	0.61	0.02	0.545	–	–	–	–	–
No	14.89 (5.97)				Ref						
**Online gaming**											
Yes	15.05 (5.68)	− 0.31	0.36	− 0.88	− 0.03	0.381	–	–	–	–	–
No	15.36 (5.42)				Ref						
**YouTubing or watching videos**											
Yes	15.31 (5.49)	0.79	0.72	1.10	0.03	0.271	–	–	–	–	–
No	14.52 (5.73)				Ref						
**Using social media like FB, Twitter, Instagram**											
Yes	15.29 (5.46)	0.51	0.71	0.72	0.02	0.470	–	–	–	–	–
No	14.78 (6.12)				Ref						
**Movie/TV series downloading**											
Yes	15.65 (5.54)	1.07	0.35	3.12	0.09	0.002	0.70	0.32	2.16	0.06	0.031
No	14.57 (5.36)				Ref					Ref	
**Online shopping**											
Yes	15.23 (5.44)	− 0.07	0.34	− 0.20	− 0.01	0.838	–	–	–	–	–
No	15.3 (5.59)				Ref						

*SD* Standard deviations; *B* unstandardized regression coefficient; *SE* Standard error; *β* standardized regression coefficient.

## Discussion

A growing body of research suggests that PUI and PUF are alarmingly increasing among young adults, particularly university students^33,36–39^. This study estimated the prevalence of PUI and PUF, and identified associated risk factors among university students in Bangladesh during the COVID-19 pandemic. This study found that place of residence, major of the study, failure in romantic relationship, time (hours) spent per day using the Internet, purpose of Internet use, and movie/TV series downloading were factors associated with both PUI and PUF.

The overall prevalence of PUI was 39.3% in the present study, which is higher than previous Bangladeshi studies conducted in the same context using a similar tool^27,40^. This discrepancy could be due to home confinement during the COVID-19 pandemic. As public health measures (i.e., lockdown) taken by the government of Bangladesh bound students to their homes^41^, the opportunity to develop addictive behaviors with Internet use has increased. A previous study conducted by Sayeed et *al*. identified that 43.8% of participants used the Internet problematically^23^. This disparity could be related to the lack of diversity in that study – only two universities were involved, while the current study has participants from over 25 universities. On the other hand, the prevalence of PUF was 37.1% in the present study, which is similar to previous Bangladeshi studies by Sayeed et *al*. (36.9%)^17^ and Mamun et *al.* (39.7%)^33^. Outside of Bangladesh, a multi-country study reported that the prevalence rates of PUI were 14% in South Korea, 19% in China, 35% in Hong Kong, 37.5% in Malaysia, 48% in Japan, 51% in the Philippines, 43.4% in Egypt, 30.8% in Czech, and 33.1% Slovak^42–44^. Prevalence rates for Facebook addiction has been identified as 47% in Malaysia^45^, 5.1% in Turkey^46^, and 26% in India^47^. These variations may be explained by several factors such as study setting, geographical location, sample size, tools used for measurements, different cutoff points, etc. Additionally, fear, worries, and anxiety related to COVID-19 are also associated with compulsive Internet use and increased use of social media^2^.

In this study, when compared to their rural counterparts, urban residents were less likely to be problematic Internet and Facebook users. This finding does not match with the previous findings from the same context in Bangladesh, in which no association was found between place of residence and PUI or PUF^17,23,33^. This may in part be due to the rapid rise of Internet access in rural Bangladesh through the initial Aspire to Innovate (a2i) program undertaken by the government of Bangladesh^41,48^. Another study in Turkey suggests that rural families employ more autocratic child-rearing practices than families from urban areas^49^, the mental health implications of which could be another reason why PUI and PUF are more common among students from rural areas.

This study did not find any prominent sex differences in PUF and PUI, which aligns with other studies^17,25^. However, in some studies, sex differences were found for excess Facebook and Internet use^37,50–52^. As this is a less researched topic, future attempts should be made to conduct exploratory qualitative research to get gender specific insights on this issue. PUF and PUI were higher in students who experienced failure in a romantic relationship than those who did not. This finding is consistent with previous studies in Bangladesh^17,23,53^. Breakdown of a close relationship magnifies poor mental health conditions^54,55^, most notably depression and insomnia^56^. This in turn may lead to Internet addiction, as it is well-established that individuals with depression and insomnia are more prone to PUI and PUF^17,23,27,33^.

This study shows that art majors used both the Internet and Facebook more than science majors. There is some evidence to suggest that students in arts and humanities fields experience more depressive and anxiety symptoms than students in science^57–59^. One explanation for this could be that sometimes students settle on art as their major because they cannot meet the more rigorous academic requirements of a science major^59^. Perceptions of inadequacy stemming from this may cause loneliness, depression, and anxiety, all of which can propel addictive behavior^40,60,61^. Further exploratory research is needed to understand why art students are at higher risk for PUI and PUF.

Previous evidence has also found that several online behaviors and activities (i.e., browsing education, watching videos/movies) are associated with PUI and PUF^17,23,27,33^. Using Facebook and the Internet for more than 4 hours per day increases the risk of PUI and PUF; this finding corroborates some previous studies^27,36^. Young adults spend much time on the Internet, especially social media for maintaining interpersonal interaction with relatives and friends, which results in elevated risk for both PUI and PUF^62,63^.

In this study, PUI was significantly associated with participants who described themselves as having a mediocre relationship with their parents. Hassan et *al.* conducted a study among young Bangladeshi adults, of which findings suggested that participants who experienced detached family relationships were more likely to suffer from Internet addiction^64^. When personal relationships are not in an ideal state, an individual’s mental health suffers, including developing addictions^54,55^.

### Limitations and strengths

This study has some critical limitations to consider when interpreting the results. The snowball sampling technique and the self-reported data are subjected to selection bias, reporting bias, and social desirability bias. Due to stigma, participants might not transparently answer sensitive questions related to failure in romantic relationships or history of substance use. The cross-sectional study design precludes extrapolation of causal factors for PUI and PUF, which warrants the necessity of further longitudinal research. Also, it has failed to capture data from other genders, transgenders which limits the inclusivity aspect of the study. Finally, this study did not include COVID-19 specific variables that might influence PUI and PUF. Despite these limitations, this study is among emerging research exploring PUI and PUF during the COVID-19 pandemic in Bangladesh and is critical to understanding the nature of Internet use in rapidly digitizing, low-resource settings like Bangladesh.

## Conclusions

This study showed that a substantial proportion of students scored high for PUI and PUF during the COVID-19 pandemic. Rural residence, study major, longer use, Internet use for recreational purposes other than education, downloading movies/ TV series, and failure in a romantic relationship were identified as predictors of both PUI and PUF. Targeted awareness programs focused on these factors should be tested to see whether they would mitigate PUI and PUF among young students. Psychosocial education campaigns and other appropriate academic institution-led interventions need to quickly be disseminated to prevent further excessive Internet use as the impacts of the pandemic are long lasting.

## Supplementary Information

Supplementary Information.

## Data Availability

The datasets and questionnaire used and/or analyzed during the current study will be shared upon request to corresponding author.
